# Sociodemographic disparities in chemotherapy and hematopoietic cell transplantation utilization among adult acute lymphoblastic and acute myeloid leukemia patients

**DOI:** 10.1371/journal.pone.0174760

**Published:** 2017-04-06

**Authors:** Brice Jabo, John W. Morgan, Maria Elena Martinez, Mark Ghamsary, Matthew J. Wieduwilt

**Affiliations:** 1 Loma Linda University School of Public Health, Loma Linda, California, United States of America; 2 University of California San Diego, Moores Cancer Center, La Jolla, California, United States of America; University of Kentucky, UNITED STATES

## Abstract

**Introduction:**

Identifying sociodemographic disparities in chemotherapy and hematopoietic cell transplantation (HCT) utilization for acute lymphoblastic leukemia (ALL) and acute myeloid leukemia (AML) may improve survival for underserved populations. In this study, we incorporate neighborhood socioeconomic status (nSES), marital status, and distance from transplant center with previously studied factors to provide a comprehensive analysis of sociodemographic factors influencing treatments for ALL and AML.

**Methods:**

Using the California Cancer Registry, we performed a retrospective, population-based study of patients ≥15 years old with ALL (n = 3,221) or AML (n = 10,029) from 2003 through 2012. The effect of age, sex, race/ethnicity, marital status, nSES, and distance from nearest transplant center on receiving no treatment, chemotherapy alone, or chemotherapy then HCT was analyzed.

**Results:**

No treatment, chemotherapy alone, or chemotherapy then HCT were received by 11%, 75%, and 14% of ALL patients and 36%, 53%, and 11% of AML patients, respectively. For ALL patients ≥60 years old, HCT utilization increased from 5% in 2005 to 9% in 2012 (p = 0.03). For AML patients ≥60 years old, chemotherapy utilization increased from 39% to 58% (p<0.001) and HCT utilization from 5% to 9% from 2005 to 2012 (p<0.001). Covariate-adjusted analysis revealed decreasing relative risk (RR) of chemotherapy with increasing age for both ALL and AML (trend p <0.001). Relative to non-Hispanic whites, lower HCT utilization occurred in Hispanic [ALL, RR = 0.80 (95% CI = 0.65–0.98); AML, RR = 0.86 (95% CI = 0.75–0.99)] and non-Hispanic black patients [ALL, RR = 0.40 (95% CI = 0.18–0.89); AML, RR = 0.60 (95% CI = 0.44–0.83)]. Compared to married patients, never married patients had a lower RR of receiving chemotherapy [ALL, RR = 0.96 (95% CI = 0.92–0.99); AML, RR = 0.94 (95% CI = 0.90–0.98)] or HCT [ALL, RR = 0.58 (95% CI = 0.47–0.71); AML, RR = 0.80 (95% CI = 0.70–0.90)]. Lower nSES quintiles predicted lower chemotherapy and HCT utilization for both ALL and AML (trend p <0.001).

**Conclusions:**

Older age, lower nSES, and being unmarried predicted lower utilization of chemotherapy and HCT among ALL and AML patients whereas having Hispanic or black race/ethnicity predicted lower rates of HCT. Addressing these disparities may increase utilization of curative therapies in underserved acute leukemia populations.

## Introduction

Acute myeloid leukemia (AML) and acute lymphoblastic leukemia (ALL) are potentially curable malignancies using multi-agent chemotherapy with or without hematopoietic cell transplantation (HCT). For most patients with AML, and many with ALL, chemotherapy alone is insufficient to produce a high likelihood of long-term remission and allogeneic HCT offers the best hope for cure [1–7]. Delays in initial chemotherapy and administration of HCT can adversely affect outcomes by increasing the risk of complications and relapse [8]. Additionally, lack of access to allogeneic HCT deprives some patients of this potentially curative therapy [9,10]. Identifying actionable sociodemographic factors that predict lack of access to chemotherapy and allogeneic HCT could extend these curative therapies to more patients diagnosed with acute leukemia.

Independent roles for sociodemographic predictors of chemotherapy and HCT among acute leukemia patients older than age 15 years remain unclear [11,12]. In univariable analyses, Bierenbaum *et al*. reported lower treatment utilization for AML among black patients, who were also younger, had lower median income and were less likely to have participated in clinical trials than whites [13]. Multivariable analyses of HCT utilization among acute leukemia patients using hospital discharge data for 1988 and 1991 showed that odds of HCT for acute leukemia were negatively influenced by older age, black race, self-pay status, Medicaid insurance, and HMO insurance^10^. In contrast, a large study using Texas hospital discharge data from 1999 found no difference in utilization of HCT based on race or insurance status [14]. Evaluating more recent data, Joshua *et al*. reported independently reduced odds of allogeneic, but not autologous, HCT among black AML patients relative to Caucasian AML patients, with no racial difference evident for ALL. Additionally, males experienced independently reduced odds, compared to females, for autologous and allogeneic HCT among AML patients but only for autologous HCT among ALL patients [15]. A recent California Cancer Registry study of 11, 084 patients diagnosed with AML found that, without adjustment for socioeconomic status, black race was associated with reduced odds of receiving chemotherapy while black race and Hispanic ethnicity were associated with reduced odds of HCT [16].

Decreased utilization of allogeneic HCT in black acute leukemia patients has been largely attributed to donor availability. Approximately 93 percent of non-Hispanic white leukemia patients having a compatible adult donor [17] whereas 58, 82 and 77 percent of black, Hispanic and Asian/Pacific Islander patients, respectively, have compatible donors [17]. Availability of cord blood and, more recently, increased use of haploidentical donors have contributed to increased percentage of patients undergoing hematopoietic cell transplantations [18–20]. Allogeneic HCT treatment of AML and ALL has historically been limited to patients under age 60 years but the development of reduced-intensity conditioning regimens over the past two decades has reduced treatment-related mortality and expanded the use of allogeneic HCT to older patients [21–23]. Given alternative stem cell sources and reduced-intensity conditioning chemotherapy regimens, it is no longer clear whether restrictions in donor availability or advanced recipient age should persist as barriers to allogeneic HCT. While investigators have evaluated age, race, insurance status [10] and sex [15] as predictors of HCT, socioeconomic status, marital status, and distance to a transplant center may also influence chemotherapy and HCT utilization. In addition, the impact of age on the rates of chemotherapy and HCT for ALL and AML may be changing over the last 15 years.

The objective of this study is to identify sociodemographic disparities in chemotherapy and HCT utilization among California residents diagnosed with ALL and AML from 2003 through 2012. The study was conducted using population-based California Cancer Registry data, which include sociodemographic factors as well as morphologic definition of the precise cancer type and cancer-directed therapies.

## Methods

### Case selection

The statewide CCR research database was utilized to conduct a historical (retrospective) investigation assessing disparities in chemotherapy and HCT treatment utilization by preexisting sociodemographic characteristics among non-pediatric acute leukemia patients from 2003 through 2012. The CCR is a state mandated cancer surveillance system composed of the three most populated Surveillance Epidemiology and End Results (SEER) registries of the United States [24]. CCR has collected uniform data on acute leukemia occurrence with cases classified using the International Classification of Diseases for Oncology third edition [25] since 2001. The study start and closing dates were selected based on data availability. Reporting of HCT was not systematically available before 2003 and complete data are not currently available after 2012. Acute lymphocytic leukemia cases were defined using the International Classification of Diseases for Oncology third edition (ICD–O-3) codes ranging from M-9811-9818, 9828 and M-9835-9837 for ALL, with AML cases defined by ICD codes M-9840, 9861, 9865, 9867, 9869, 9871–9874, 9891, 9895–9897, 9910, 9911 and M-9920 [25]. Favorable-risk AML was defined as M-9871, AML with inv(16)(p13.1q22) or t(16,16) (p13.1;q22); CBFB-MYH11 (M-9871) and AML with t(8;21)(q22;q22); RUNX1-RUNX1T1 (M-9896). All other AML subgroups were defined as unfavorable-risk AML. Cases of acute promyelocytic leukemia (M-9866) were excluded.

### Study outcomes

Endpoints included cancer-directed chemotherapy status (Yes *vs*. No) and HCT (Yes *vs*. No). Patients were classified as receiving chemotherapy if chemotherapy was administered alone or prior to HCT and HCT was defined as hematopoietic cell transplantation following chemotherapy.

### Study covariates

Sociodemographic characteristics included four mutually exclusive categories of race/ethnicity (Asian/other, Hispanic, non-Hispanic black, non-Hispanic white) with Asian/other race category combining patients of Asian, Pacific island, American Indian and unknown race/ethnic group origin. In addition, a multidimensional neighborhood quintile socioeconomic status (nSES) index based on place of residence at diagnosis was also included in the analyses. The ecologic SES index used in this study was derived elsewhere [26,27] using seven census derived economic variables measured at the block group level covering two time-periods (2003–2005 and 2006–2012) [26,27]. nSES index variables included education, median income, percentage living below the poverty level, median rent, median house value, proportion with a blue-collar job and proportion in the workforce without a job that were older than age 16 years [26,27]. As described by Yost et al. [26], and updated by Yang et al. [27], these seven element variables were included in a principal component analysis that generated standardized SES index scores for each of the more than 20,000 census block groups in California. In a second phase, these standardized scores were sorted and grouped into equal range quintile categories in which a value of one represented the lowest nSES category. Other covariates in the analyses included age at diagnosis (age), sex, marital status as single, married, DSW (divorced, separated, or widowed), residence distance from the nearest transplant center (<50, 50–99 and 100+ miles), year of diagnosis and cytogenetic risk groups as favorable *versus* unfavorable group.

### Statistical analysis

Chemotherapy and HCT treatment among ALL and AML patients were common events, ensuring that odds ratios for chemotherapy and HCT would overestimate relative risk [28]. Log-binomial and negative binomial regression models did not converge, therefore Poisson regression with robust confidence intervals [29,30] was used to estimate relative risk (RR) of treatment with 95 percent confidence interval limits. In addition, two way interactions for binary age (< 60 years and > = 60 years), sex or SES as low SES (1st-3rd SES quintiles as low SES and 4^th^ and 5^th^ SES quintiles as high SES) with marital status (Yes *vs*.No) were conducted. The Cochran-Armitage test for trend was used to assess changes in chemotherapy or HCT utilization over the 10-year study-period for both AML and ALL patients. Predictors included in analyses were selected *a priori* based on previously published studies.

Google maps API [31] was used to compute the shortest driving distance between place of residence and any of 11 transplant centers in California (Alta Bates Summit Medical Center, Cedars-Sinai Medical Center, City of Hope National Medical Center, Loma Linda University Medical Center, Scripps Green Hospital, Stanford Health Care, Sutter Medical Center Sacramento, University of California San Diego Medical Center, University of California San Francisco Medical Center, University of California Los Angeles and University of California Davis Medical Center).

Crude and adjusted relative risks for chemotherapy and HCT (Yes *vs*. No) were computed using robust Poisson regression with separate analyses performed for ALL and AML patients. Model fitness assumption was assessed using Pearson’s chi-squared tests did not reveal over dispersion. We also checked for multicollinearity and there was no evidence of highly correlated covariates. Influence analysis did not identify the presence of any influential observations or critical outliers [32,33]. Using the 10 cases per variable level included in analyses, showed that we had enough sample size for analyses, furthermore, power analysis confirmed that we had 80 percent or more power for all our analytical sample sizes using effect size of RR = 1.20 or greater. All statistical tests were two-side and conducted at a significance level of 0.05. Statistical analyses were performed using R [34] and SAS/STAT software, Version 9.4 of the SAS System for Windows. (Copyright © 2002–2012 SAS Institute Inc. SAS and all other SAS Institute Inc. product or service names are registered trademarks or trademarks of SAS Institute Inc., Cary, NC, USA).

## Results

### Study population

**Fig 1:**
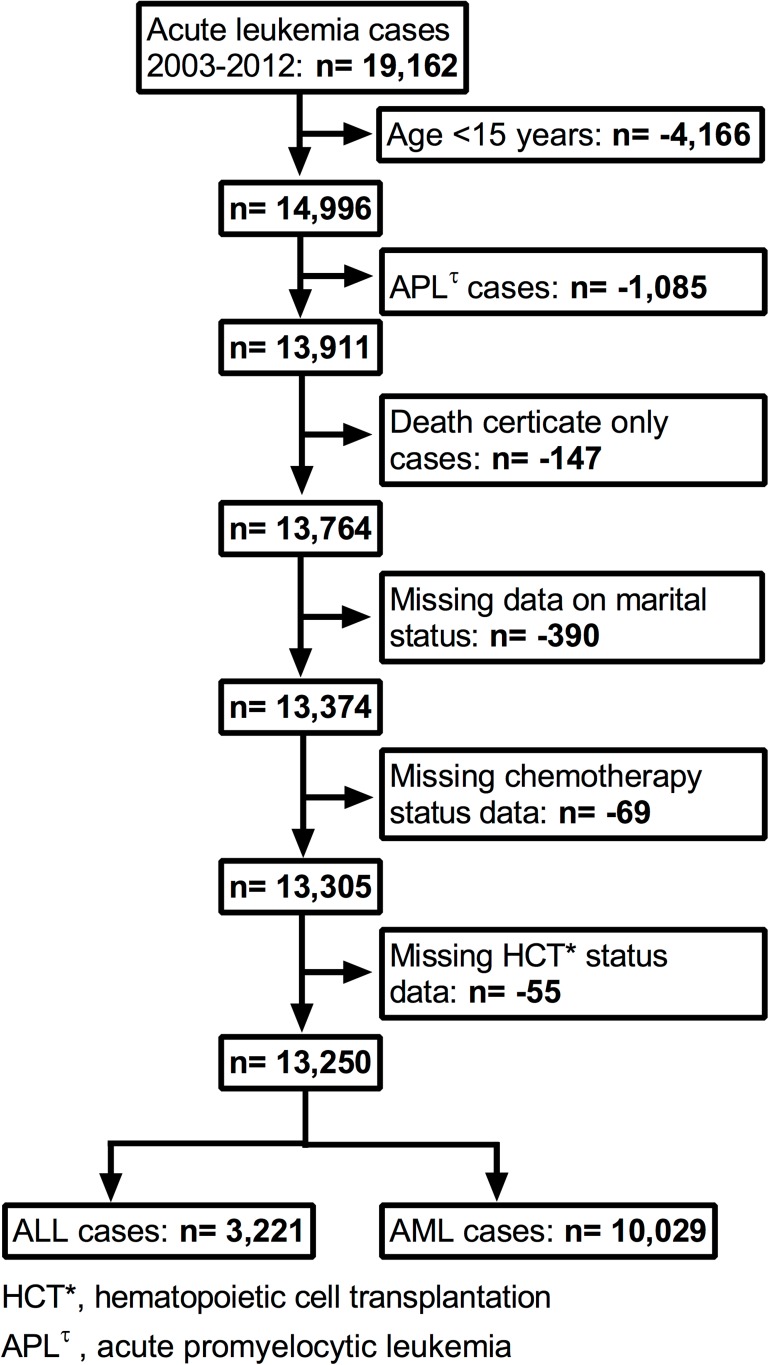
Study patient selection flowchart for acute myeloid (AML) and acute lymphocytic leukemia (ALL) cases.

Data for 19,162 California residents diagnosed with ALL or AML between January 2003 and December 2012 were extracted from the CCR research database. Patients below age 15 years (n = 4,166) were excluded from study, as were patients with acute promyelocytic leukemia (M-9866, n = 1,085), having death certificate only diagnosis (n = 147), and missing marital status or place of residence information (n = 390). In addition 69 patients with unknown chemotherapy status and 55 patients missing information on HCT were excluded from analysis. The remaining 13,250 patients included 3,221 diagnosed with ALL and 10,029 with AML (Fig 1).

#### Demographic and treatment patterns

Approximately 76% of ALL and 32% of AML patients were age less than 60 years at diagnosis. Females represented 43% of ALL and 45% of AML patients. Among Hispanics, ALL represented 47.3% of all acute leukemia cases compared 20.5%, 19.0% and 15.6% of acute leukemia cases among Asian/other, non-Hispanic blacks, and NHWs, respectively. There was a tendency for lower nSES among ALL patients, with the opposite pattern seen for AML (Table 1). Patients diagnosed with ALL were more likely to have received chemotherapy compared to AML patients (88.6% and 63.5%, respectively). Utilization of HCT was similar for ALL (13.9%) and AML (11%). Patients age 15–39 years had the highest HCT utilization among ALL patients (51.7%) while patients age 40–59 years had the highest HCT utilization among AML patients (49%). In addition, only 3.4% of ALL patients and 3.6% of AML patients over age 60 underwent HCT during the study period (Table 1).

**Table 1:** Sociodemographic characteristics for acute lymphoblastic leukemia and acute myeloid leukemia cases by treatment as no chemotherapy (None), chemotherapy alone, or chemotherapy and hematopoietic cell transplantation (HCT) in California, 2003–2012.

	Acute Lymphoblastic Leukemia			Acute Myeloid Leukemia		
	Treatment, count (%)			Treatment, count (%)		
Population Characteristic	None	Chemotherapy alone	HCT	None	Chemotherapy alone	HCT
**N**	367	2,405	449	3,657	5,266	1,106
**Age**						
15–39	58 (15.8)	1,251 (52.0)	232 (51.7)	94 (2.6)	596 (11.3)	314 (28.4)
40–59	58 (15.8)	644 (26.8)	190 (42.3)	294 (8.0)	1,319 (25.1)	542 (49.0)
60 +	251 (68.4)	510 (21.2)	27 (6.0)	3,269 (89.4)	3,351 (63.6)	250 (22.6)
**Sex**						
Male	194 (52.9)	1,403 (58.3)	256 (57.0)	1,960 (53.6)	2,986 (56.7)	550 (49.7)
Female	173 (47.1)	1,002 (41.7)	193 (43.0)	1,697 (46.4)	2,280 (43.3)	556 (50.3)
**Race/Ethnicity**						
Asian/Other^**¥**^	33 (9.0)	224 (9.3)	60 (13.4)	390 (10.7)	668 (12.7)	172 (15.6)
Hispanic	126 (34.3)	1,255 (52.2)	199 (44.3)	497 (13.6)	1,029 (19.5)	229 (20.7)
Non-Hispanic black	22 (6.0)	96 (4.0)	6 (1.3)	197 (5.4)	299 (5.7)	34 (3.1)
Non-Hispanic white	186 (50.7)	830 (34.5)	184 (41.0)	2,573 (70.4)	3,270 (62.1)	671 (60.7)
**Marital status**						
Never married	96 (26.2)	1,137 (47.3)	166 (37.0)	462 (12.6)	1,044 (19.8)	287 (26.0)
Married	171 (46.6)	1,014 (42.2)	244 (54.3)	1,894 (51.8)	3,091 (58.7)	701 (63.4)
DSW*	100 (27.3)	254 (10.5)	39 (8.7)	1,301 (35.6)	1,131 (21.5)	118 (10.7)
**Distance to nearest transplant center**						
< 50 Miles	261 (71.1)	1,778 (74.0)	342 (76.2)	2,670 (73.0)	3,814 (72.4)	821 (74.2)
50–99 Miles	59 (16.1)	324 (13.5)	58 (12.9)	588 (16.1)	779 (14.8)	170 (15.4)
100 + Miles	47 (12.8)	303 (12.5)	49 (10.9)	399 (10.9)	673 (12.8)	115 (10.4)
**nSES**						
Lowest	82 (22.3)	678 (28.2)	90 (20.0)	506 (13.8)	816 (15.5)	116 (10.5)
2	94 (25.6)	508 (21.1)	84 (18.7)	717 (19.6)	987 (18.7)	163 (14.7)
3	72 (19.6)	456 (19.0)	76 (16.9)	755 (20.7)	1,045 (19.8)	212 (19.2)
4	61 (16.6)	414 (17.2)	103 (22.9)	876 (24.0)	1,170 (22.2)	281 (25.4)
Highest	58 (15.8)	349 (14.5)	96 (21.4)	803 (22.0)	1,248 (23.7)	334 (30.2)
**Year of diagnosis**						
2003–2004	66 (18.0)	426 (17.7)	61 (13.6)	725 (19.8)	817 (15.5)	165 (14.9)
2005–2006	60 (16.4)	441 (18.3)	79 (17.6)	705 (19.3)	878 (16.7)	196 (17.7)
2007–2008	59 (16.1)	466 (19.4)	92 (20.5)	715 (19.6)	1,036 (19.7)	217 (19.6)
2009–2010	87 (23.7)	516 (21.5)	81 (18.0)	751 (20.5)	1,162 (22.1)	238 (21.5)
2011–2012	95 (25.9)	556 (23.1)	136 (30.3)	761 (20.8)	1,373 (26.1)	290 (26.2)
**Cytogenetics**						
Unfavorable	-	-	-	3,608 (98.7)	5,041 (95.7)	1,050 (94.9)
Favorable	-	-	-	49 (1.3)	225 (4.3)	56 (5.1)

^**¥**^Asian/Others includes patients of Asian descent including Filipino, other includes American Indian, Native Alaskan, Mixed or unknown race/ethnicity.

*Divorced, separated, or widowed.

#### Effect of age

Covariate adjusted chemotherapy findings among acute leukemia patients (Table 2), showed a decreasing RR of chemotherapy with increasing age among ALL (trend p <0.001) and AML patients (trend p <0.001). Using age group 40–59 as the referent category, covariate adjusted RR for receiving chemotherapy among patients age 15–24 years was 1.08 (95% CI = 1.04–1.12) for ALL and 1.10 (95% CI = 1.04–1.17) for AML patients, while RR for chemotherapy among patients age 60–75 years at diagnosis was 0.86 (95% CI = 0.82–0.91) for ALL and 0.83 (95% CI = 0.80–0.86) for AML (Table 2). The covariate adjusted age effect for HCT among patients age 15–39 years was 0.99 (95% CI = 0.81–1.20) for ALL and 1.37 (95% CI = 1.21–1.54) for AML. Among patients age 60 years and older, the adjusted RR for HCT was 0.20 (95% CI = 0.14–0.29; Table 3) for ALL and 0.23 (95% CI = 0.20–0.26) for AML (Table 3).

**Table 2:** Unadjusted and adjusted^†^ relative risks (RR) associations between sociodemographic characteristics and chemotherapy administration (Yes *versus* No) in California, 2003–2012.

	Acute Lymphoblastic Leukemia					Acute Myeloid Leukemia				
	Unadjusted		Adjusted^†^			Unadjusted		Adjusted^†^		
Population Characteristic	RR	95% CI^±^	RR	95% CI^±^	Trend p-value	RR	95% CI^±^	RR	95% CI^±^	Trend p-value
**Age**					<0.001					<0.001
15–24	1.05	1.02–1.08	1.08	1.04–1.12		1.08	1.03–1.13	1.10	1.04–1.17	
25–39	1.03	1.00–1.06	1.03	1.00–1.07		1.04	0.99–1.09	1.05	1.00–1.10	
40–59	1		1			1		1		
60–75	0.87	0.82–0.91	0.86	0.82–0.91		0.83	0.81–0.86	0.83	0.80–0.86	
76+	0.50	0.44–0.57	0.50	0.44–0.57		0.43	0.41–0.45	0.43	0.41–0.46	
**Sex**					NA					NA
Male	1		1			1		1		
Female	0.97	0.94–1.00	1.01	0.98–1.03		0.95	0.92–0.98	0.97	0.94–1.00	
**Race/Ethnicity**					NA					NA
Asian/Other^**¥**^	1.07	1.01–1.13	1.01	0.96–1.06		1.13	1.07–1.19	1.04	0.99–1.09	
Hispanic	1.11	1.08–1.15	1.02	0.98–1.05		1.20	1.16–1.26	1.03	0.98–1.07	
Non-Hispanic black	1.00	0.91–1.09	0.96	0.88–1.04		1.08	1.00–1.16	0.98	0.91–1.05	
Non-Hispanic white	1		1			1		1		
**Marital status**					NA					NA
Never married	1.08	1.05–1.11	0.96	0.92–0.99		1.12	1.07–1.16	0.94	0.90–0.98	
Married	1		1			1		1		
DSW*	0.84	0.78–0.90	0.97	0.91–1.03		0.75	0.71–0.79	0.91	0.87–0.95	
**Distance to nearest transplant center**					0.62					0.12
< 50 Miles	1		1			1		1		
50–99 Miles	0.97	0.93–1.02	0.97	0.93–1.01		0.97	0.92–1.02	1.00	0.95–1.04	
100 + Miles	0.99	0.95–1.04	1.00	0.96–1.04		1.07	1.01–1.12	1.05	1.00–1.10	
**nSES**					0.001					<0.001
Lowest	1.04	0.99–1.09	0.95	0.90–0.99		1.01	0.96–1.07	0.89	0.84–0.94	
2	0.98	0.93–1.04	0.92	0.87–0.97		0.95	0.90–1.00	0.89	0.85–0.93	
3	1.01	0.96–1.06	0.96	0.92–1.01		0.95	0.91–1.01	0.91	0.87–0.95	
4	1.02	0.96–1.07	1.00	0.95–1.05		0.94	0.89–0.99	0.91	0.87–0.95	
Highest	1		1			1		1		
**Year of diagnosis**					0.58					<0.001
2003–2004	1		1			1		1		
2005–2006	1.02	0.97–1.07	1.01	0.97–1.05		1.05	0.98–1.12	1.07	1.01–1.13	
2007–2008	1.03	0.98–1.07	1.03	0.99–1.07		1.12	1.05–1.19	1.12	1.06–1.18	
2009–2010	0.99	0.94–1.04	0.99	0.95–1.04		1.15	1.08–1.22	1.16	1.10–1.23	
2011–2012	0.99	0.94–1.03	1.00	0.96–1.04		1.21	1.15–1.29	1.22	1.16–1.29	
**Cytogenetics**					NA					NA
Unfavorable	-	-	-	-		1		1		
Favorable	-	-	-	-		1.41	1.33–1.49	1.15	1.09–1.22	

^†^Adjusted for each of the other covariates listed in Table 2.

^± ^95% CI represents lower and upper 95 percent confidence interval limits for relative risks.

NA, not applicable because trend test requires three or more ordinal exposure levels.

^**¥**^Asian/Others includes patients of Asian descent including Filipino, other includes American Indian, Native Alaskan, Mixed or unknown race/ethnicity.

*Divorced, separated, or widowed.

**Table 3:** Unadjusted and adjusted^†^ relative risks (RR) associations between sociodemographic characteristics and hematopoietic cell transplantation versus chemotherapy alone in California, 2003–2012.

	Acute Lymphoblastic Leukemia					Acute Myeloid Leukemia				
	Unadjusted		Adjusted^†^			Unadjusted		Adjusted^†^		
Population Characteristic	RR	95% CI^±^	RR	95% CI^±^	Trend p-value	RR	95% CI^±^	RR	95% CI^±^	Trend p-value
**Age**					<0.001					<0.001
15–39	0.69	0.58–0.82	0.99	0.81–1.20		1.18	1.06–1.33	1.37	1.21–1.54	
40–59	1		1			1		1		
60 +	0.22	0.15–0.33	0.20	0.14–0.29		0.24	0.21–0.27	0.23	0.20–0.26	
**Sex**					NA					NA
Male	1		1			1		1		
Female	1.05	0.88–1.24	1.06	0.89–1.25		1.26	1.13–1.40	1.15	1.04–1.28	
**Race/Ethnicity**					NA					NA
Asian/Other^**¥**^	1.16	0.90–1.51	1.08	0.84–1.39		1.20	1.04–1.40	0.97	0.84–1.11	
Hispanic	0.75	0.63–0.91	0.80	0.65–0.98		1.07	0.93–1.22	0.86	0.75–0.99	
Non-Hispanic black	0.32	0.15–0.71	0.40	0.18–0.89		0.60	0.43–0.83	0.60	0.44–0.83	
Non-Hispanic white	1		1					1		
**Marital status**					NA					NA
Never married	0.66	0.55–0.79	0.58	0.47–0.71		1.17	1.03–1.32	0.80	0.70–0.90	
Married	1		1			1		1		
DSW*	0.69	0.50–0.94	0.94	0.69–1.27		0.51	0.43–0.61	0.70	0.59–0.84	
**Distance to nearest transplant center**					0.53					0.38
< 50 Miles	1		1			1		1		
50–99 Miles	0.94	0.73–1.22	0.91	0.71–1.17		1.01	0.87–1.17	1.11	0.96–1.28	
100 + Miles	0.86	0.65–1.14	0.95	0.71–1.25		0.82	0.69–0.99	0.87	0.73–1.04	
**nSES**					<0.001					<0.001
Lowest	0.54	0.42–0.71	0.63	0.47–0.84		0.59	0.49-.72	0.52	0.43–0.64	
2	0.66	0.5–0.86	0.71	0.54–0.94		0.67	0.57–0.80	0.60	0.51–0.71	
3	0.66	0.5–0.87	0.69	0.53–0.91		0.80	0.68–0.93	0.73	0.63–0.85	
4	0.92	0.72–1.18	0.98	0.77–1.25		0.92	0.80–1.06	0.85	0.75–0.97	
Highest	1		1			1		1		
**Year of diagnosis**					<0.001					0.02
2003–2004	1		1			1		1		
2005–2006	1.21	0.89–1.65	1.30	0.96–1.76		1.09	0.9–1.31	1.11	0.93–1.33	
2007–2008	1.32	0.98–1.78	1.47	1.10–1.97		1.03	0.86–1.24	1.05	0.89–1.25	
2009–2010	1.08	0.79–1.48	1.22	0.90–1.66		1.01	0.84–1.21	1.17	0.98–1.38	
2011–2012	1.57	1.19–2.07	1.76	1.34–2.30		1.04	0.87–1.24	1.22	1.03–1.43	
**Cytogenetics**					NA					NA
Unfavorable	-	-	-	-		1		1		
Favorable	-	-	-	-		1.16	0.91–1.47	0.82	0.64–1.04	

^†^Adjusted for each of the other covariates listed in Table 3.

^± ^95% CI represents lower and upper 95 percent confidence interval limits for relative risks.

NA, not applicable because trend test requires three or more ordinal exposure levels.

^**¥**^Asian/Others includes patients of Asian descent including Filipino, other includes American Indian, Native Alaskan, Mixed or unknown race/ethnicity.

*Divorced, separated, or widowed.

#### Effect of sex

Chemotherapy utilization among females, compared to males, showed an RR of 1.01 (95% CI = 0.98–1.03) for ALL and 0.97 (95% CI = 0.94–1.00) for AML (Table 2). HCT utilization among females showed a RR of 1.06 (95% CI = 0.89–1.25) for ALL and 1.15 (95% CI = 1.04–1.28) for AML (Table 3).

#### Effect of race/ethnicity

Evaluations of chemotherapy utilization comparing each race/ethnic group with NHWs showed RRs near unity (Table 2). Covariate adjusted relative risks among ALL patients contrasting HCT utilization for Asian/others, Hispanics, and non-Hispanic blacks with NHWs were RR = 1.08 (95% CI = 0.84–1.39), 0.80 (95% CI = 0.65–0.98) and 0.40 (95% CI = 0.18–0.89), respectively (Table 3). Similar contrasts for AML showed adjusted RRs for HCT of 0.97 (95% CI = 0.84–1.11), 0.86 (95% CI = 0.75–0.99) and 0.60 (95% CI = 0.44–0.83) for Asian/others, Hispanics, and non-Hispanic blacks, respectively, when contrasted with NHWs (Table 3). Analyses that excluded patients of American Indian and unknown race/ethnic groups produced estimates for Asian/Pacific Islander (results not shown) that were very similar to those obtained with the Asian/other classification.

#### Effect of marital status

**Fig 2:**
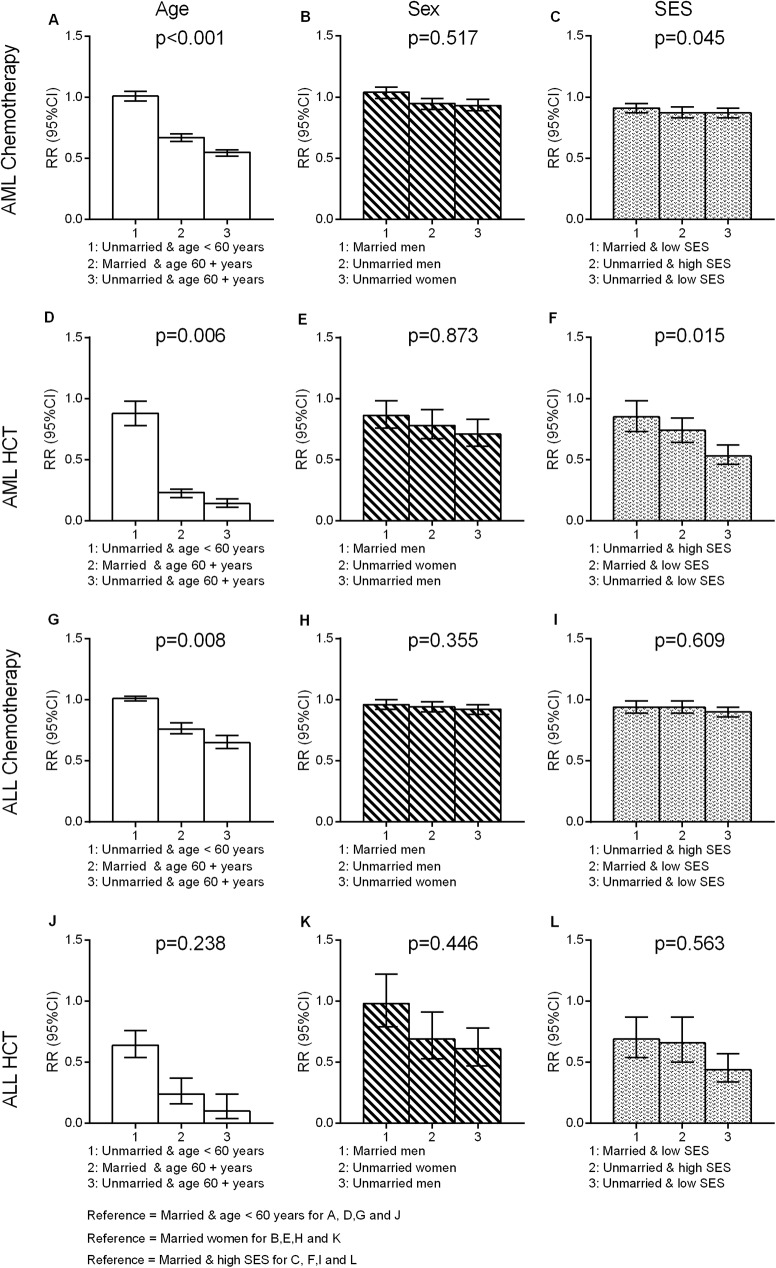
Evaluation of interaction of age, sex or SES by marital status as a predictor of treatment for AML and ALL patients.

Contrasted with married ALL patients, patients who were never married or divorced, separated, or widowed at diagnosis showed RRs for chemotherapy of 0.96 (95% CI = 0.92–0.99) and 0.97 (95% CI = 0.91–1.03), respectively (Table 2). Contrasts of marital status for AML patients showed RRs of 0.94 (95% CI = 0.90–0.98) for never married and 0.91 (95% CI = 0.87–0.95) for divorced, separated, or widowed patients, as compared with married patients. Marital status contrasts for HCT utilization among ALL patients showed RRs of 0.58 (95% CI = 0.47–0.71) for never married and 0.94 (95% CI = 0.69–1.27) for divorced, separated, or widowed versus married (Table 3). Similarly, contrasts for HCT utilization among AML patients showed RRs of 0.80 (95% CI = 0.70–0.90) for never married and 0.70 (95% CI = 0.59–0.84) for divorced, separated, or widowed versus married patients (Table 3). Lower use of HCT among was particularly prominent among unmarried patients living in low SES neighborhoods (Fig 2F and 2L) and unmarried patients over the age of 60 years (Fig 2D and 2J).

#### Effect of distance from transplant center

Compared to distance less than 50 miles, residence distance of 50–99 miles showed RRs of 0.97 (95% CI = 0.93–1.01) and 1.00 (95% CI = 0.95–1.04) for ALL and AML patients, respectively; while residence distance of 100 miles or greater showed RR of 1.00 (95% CI = 0.96–1.04) and 1.05 (95% CI = 1.00–1.10) for ALL and AML patients, respectively (Table 2). Contrasts for HCT utilization showed RRs of 0.91 (95% CI = 0.71–1.17) and 1.11 (95% CI = 0.96–1.28) for ALL and AML patients residing within 50 miles of a transplant center, respectively (Table 3), while residence distance of 100 miles or greater showed RR of 0.95 (95% CI = 0.71–1.25) and 0.87(95% CI = 0.73–1.04) for ALL and AML patients respectively (Table 3).

#### Effect of neighborhood socioeconomic status

Compared with the highest nSES quintile, progressively lower SES levels independently predicted lower chemotherapy utilization for ALL (Trend p<0.001) and AML (Trend p<0.001) (Table 2). Similar findings were evident for HCT among AML (Trend p<0.001) and ALL (Trend p<0.001) patients (Table 3). For both ALL and AML, the lowest SES quintile showed a reduced relative risk for receipt of chemotherapy [ALL, RR = 0.95 (95% CI 0.90–0.99); AML, RR = 0.89 (95% CI 0.84–0.94)] and to a greater extent HCT [ALL, RR = 0.63 (95% CI 0.47–0.84); AML, RR = 0.52 (95% CI 0.43–0.64)] when contrasted with the highest SES quintile.

#### Effect of year of diagnosis

**Fig 3:**
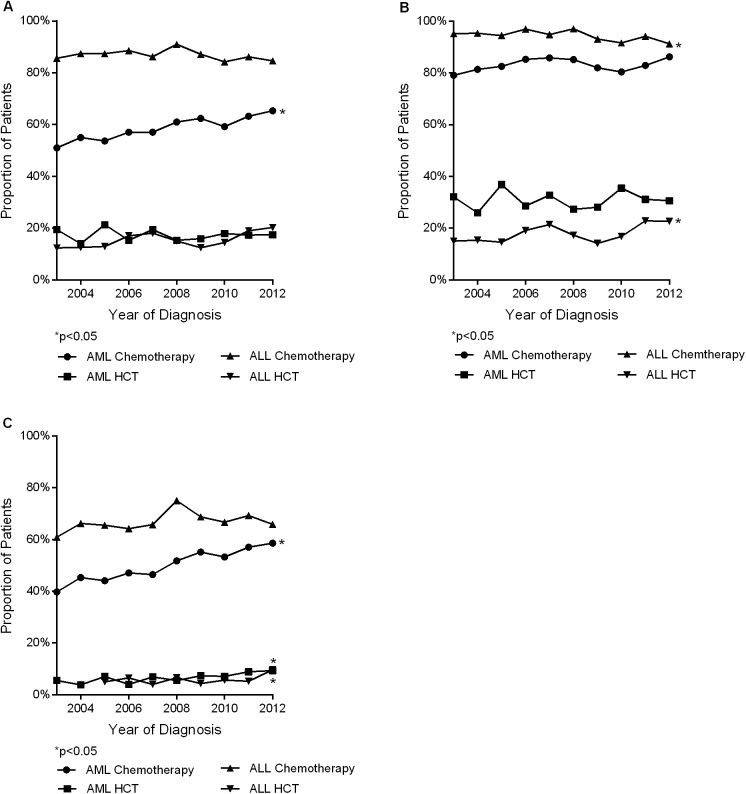
Trends for percentage of patients receiving chemotherapy or HCT treatment among acute leukemia patients in California for years 2003–2012. (A) ALL and AML patients age 15 years and older. (B) ALL and AML patients age 15–59 years. (C) ALL and AML patients age 60 years and older.

The crude percentage of AML patients receiving chemotherapy was 51 percent in 2003, climbing to 65 percent in 2012 (Trend p<0.001, Fig 3A). A similar trend for HCT utilization among ALL patients was seen: 12 percent in 2003 and 20 percent in 2012 (Trend p = 0.05, Fig 3A). No change was seen in rates of chemotherapy among ALL patients (Trend p = 0.28) and HCT among AML patients (Trend p = 0.67) over the study period (Fig 3A). For ALL patients less than 60 years of age, chemotherapy utilization declined from 95 percent in 2003 to 91 percent in 2012 (Trend p = 0.02) while HCT utilization increased from 15 to 22 percent within the same period (Fig 3B). From 2003 through 2012, AML patients less than 60 years of age showed unchanged rates if chemotherapy (Trend p = 0.22) and HCT (Trend p = 0.94, Fig 3B). In addition, for ALL patients age 60 years and older, chemotherapy utilization remained relatively flat (Trend p = 0.38), while HCT utilization increased from 5 percent in 2005 to 9 percent in 2012 (p = 0.03) (Fig 3C). Among AML patients age 60 years and older, chemotherapy utilization increased from 39 percent in 2003 to 58 percent in 2012, (p<0.001) and HCT utilization increased from 5 percent in 2003 to 9 percent in 2012 (p<0.001) (Fig 3C). Covariates adjusted biannual contrasts of chemotherapy utilization among ALL patients for years 2005–2006 through 2011–2012 with 2003–2004 showed no change (p = 0.58), However, increased chemotherapy utilization was observed among AML patients (Trend p<0.001) and increased HCT utilization among both ALL (Trend p<0.001) and AML (Trend p = 0.02) patients over the 10 year period (Tables 2 and 3).

#### Effect of cytogenetic risk group among AML patients

Compared with AML patients having unfavorable cytogenetics, AML patients with favorable cytogenetics had a RR of 1.15 (95% CI 1.09–1.22) for chemotherapy and 0.82 (95% CI 0.64–1.04) for HCT utilization (Tables 2 and 3).

## Discussion

In addition to the roles of age, sex, and race as predictors of acute leukemia treatment identified in other studies, findings reported here also assessed a multidimensional nSES index, marital status, and distance between usual place of residence and nearest transplant center. Our findings are consistent with previous studies reporting lower HCT utilization among Hispanic and non-Hispanic black acute leukemia patients [10,15,16]. Additional findings reveal that increasing SES quintile categories independently predicted increased chemotherapy and HCT utilization among both ALL and AML patients, with substantially lower use of HCT among unmarried patients with low nSES.

Differences between results presented here, that included a multidimensional SES index, and those reported by others can, in part, be attributed to failure to consider SES by previous investigators [15,16]; reliance on a single SES indicator [10,13]; combinations of pediatric, adolescent and adult acute leukemia cases; and/or analyses that combined leukemia subtypes [10,15]. Reasons for differences in therapies for ALL and AML patients classified in various nSES categories are not fully understood, although differences in disease risk, clinical trial participation [13], health insurance status, treatment referral patterns [9], treatment aggressiveness, comorbidities, patient compliance, time to diagnosis [8], and patient support system [35] may each contribute to this disparity. Unlike race/ethnicity, many nSES-related variable components may be modifiable, possibly through societal efforts to facilitate insurance coverage, transportation and housing, justifying further research to delineate actionable neighborhood SES index components.

Consistent with findings made by others [10,14], chemotherapy and HCT utilization reported here were lowest among older acute leukemia patients with further reduced treatment utilization observed among older patients that were not married. Reasons for reduced utilization of HCT in older patients may include lower suitability for chemotherapy and HCT because of poor performance status, increased acuity at presentation, and comorbidities that lower tolerance for aggressive treatment [36], although physician biases and nihilism [9] regarding the value of chemotherapy and HCT in the older population may also play a role despite growing evidence that the older population can benefit from chemotherapy and HCT.

Practices and attitudes about aggressively treating older acute leukemia patients may be changing, however. Results presented in this manuscript reveal a linear increase in chemotherapy utilization among AML patients age 60 years and older and a linear increase in HCT utilization among both ALL and AML patients age 60 years and older from 2003 to 2012 (Fig 3C). This observed improvement in treatment utilization among older acute leukemia patients may be due to changes in physician attitudes, the advent of lower intensity induction therapies, greater availability of clinical trial therapies, and the development of reduced-intensity conditioning regimens for HCT [36].

In agreement with findings previously reported by Joshua *et al*. [15], our findings reveal that females were more likely than males to receive HCT for AML, but not for ALL, with lowest HCT utilization observed among unmarried men. The reasons for this difference are unclear, although earlier diagnosis, fewer comorbidities, better tolerability of pre-transplant chemotherapy, better compliance with treatment and more extensive support systems may play a role.

Marital status may also be an indicator of a more robust support system. Marital status has been shown to predict treatment and health outcomes in solid tumors. In an analysis of 1,260,898 patients diagnosed with 10 selected cancers excluding acute leukemia between 2004 and 2008 [35], never married, separated, divorced, and widowed patients were significantly more likely to present with advanced disease, less likely to receive definitive therapy, and more likely to die from their cancer, relative to married patients. Similar to patients with solid tumors, findings in our study revealed that never-married and divorced, separated, or widowed acute leukemia patients had lower chemotherapy and HCT utilization when compared to married acute leukemia patients. Additionally, marital status appeared to modify the effects of age and nSES, with poorer treatment outcomes consistently observed among older (age 60+ years) unmarried patients and among unmarried patients living in low SES neighborhoods. Our study could not determine why married patients were more likely to receive chemotherapy and HCT relative to never-married and divorced, separated, or widowed patients, although it is possible that later disease presentation, comorbidities, poor compliance with treatment plans and lack of a dedicated caregiver could contribute to the observed difference.

In our analyses, distance between residence and nearest transplant center showed no significant predictive effect for chemotherapy and HCT utilization among ALL and AML patients. Our HCT findings are consistent with those reported by Ragon *et al*., in which residence distance from a transplant center did not predict survival for allogeneic HCT [37], challenging the relevance of a potential distance effect. It seems reasonable to conclude that the null findings found in our study for the distance variable are, in part, because patients already comply with the near-residence requirement maintained by treatment centers. Furthermore, our findings revealed that 73 percent of acute leukemia patients in California already resided within 50 miles from a transplant center at diagnosis, suggesting adherence to the near-residence requirement was easily achieved for most patients and that relocation for HCT was an uncommon event.

A linear increase in chemotherapy and HCT utilization for AML patients as well as HCT utilization for ALL patients over the 10-year study period was evident in the unadjusted findings and persisted in the covariate adjusted RR. However, age-stratified analysis showed that the observed improvement in treatment utilization over the study period was driven by growing treatment utilization among patients age 60 years and older while a slight decline in chemotherapy utilization was evident for ALL patients age less than 60 years. Our findings for AML are consistent with those reported by Medeiros *et al*., in which both studies showed improved treatment utilization among AML patients age 65 years and older [38], with our findings revealing also increased HCT utilization among ALL patients.

This study has some limitations. In spite of the sociodemographic diversity and large size of the California population, treatment patterns and proximity to treatment centers for acute leukemia may vary regionally, limiting generalizability of these findings to other U.S. and international populations. California specifically has a diverse racial/ethnic population [39], a large percentage of population in urban areas [40], a higher median income [41], and differing access to health insurance including Medicaid than some states [42]. This study reveals that socio-demographic factors, including the CCR ecologic SES index, predict utilization of chemotherapy and HCT among acute leukemia patients, although it does not distinguish between individual and potentially actionable characteristics included in the CCR nSES index. Although age, sex, race/ethnicity and marital status each could correlate with our ecologic nSES index, these demographic variables are not included in the index and have exhibited sufficient independence to distinguish their effects from the SES index in other studies [43,44]. Furthermore, multicolinearity between age, sex, race/ethnicity or marital status and our ecologic SES index was not detected in this dataset. Insurance status is also not included as a variable in this study. Previous research has challenged the validity of the CCR insurance (payer source) data [45]. Additionally, several of the element variables included in the CCR nSES index, and other demographic variables in our analysis models, would reasonably be expected to correlate with insurance status. To ensure validity of our findings, payer source was not included in our analyses.

Using CCR data from 1996–1997, Ayanian *et al*. cited underreporting of adjuvant chemotherapy in 13 percent of stage III colorectal cancer patients [46]. Our assessments of 2003–2012 data revealed that 99.5 percent of acute leukemia patients had reports of either “Yes” or “No” in the treatment field for chemotherapy and 99.6 percent for HCT in the current CCR research database. Although missing data has potential to introduce systematic error, the level of completeness in chemotherapy and HCT treatment variables in the current CCR database for acute leukemia patients minimizes this potential. Furthermore, multiple imputations for chemotherapy and HCT did not significantly alter our findings (results not shown). Our assessment of distance between current residence and nearest transplant center presumes that all patients would be equally eligible for HCT in each of the centers. It seems reasonable to assume that bias introduced by this assumption would tend to underestimate distance to the preferred transplant center for some acute leukemia patients, although no mechanism was available to measure or correct this potential bias.

## Conclusions

Our findings reveal that, in addition to age, nSES and marital status were independent and dose-related predictors of chemotherapy and HCT utilization acute leukemia patients. Unlike findings from other researchers, race/ethnicity and sex did not represent barriers to chemotherapy among acute leukemia patients in the diverse and contemporary California population. Although Hispanic and non-Hispanic black race/ethnicity were not independent predictors of chemotherapy among acute leukemia patients, Hispanic and non-Hispanic black race/ethnicity persisted as independent negative predictors of HCT utilization. Married patients had higher utilization of both chemotherapy and HCT relative to unmarried patients when adjusted for other factors. These findings support a shift from research and interventions addressing chiefly racial and ethnic barriers to care for acute leukemia to also addressing barriers raised by socioeconomic disparities, advanced age, and unmarried status. Better understanding and correction of specific actionable factors within each of these underserved populations should lead to more equitable and inclusive access to potentially curative chemotherapy and hematopoietic cell transplantation for ALL and AML patients.
